# Investigation of ocular biometry in 4– to 9-year-old Chinese children

**DOI:** 10.1186/s12886-023-02975-5

**Published:** 2023-05-19

**Authors:** Ming-Hui Zhao, Yi Song, Jia-li Liu, Juan Li, Ying Wang, Yan-jun Hua, Qiang Wu

**Affiliations:** 1 Department of Ophthalmology, Shanghai Municipal Hospital of Traditional Chinese Medicine, Shanghai University of Traditional Chinese Medicine, Shanghai, 200071 China; 2 Department of Ophthalmology, Shanghai Jiaotong University Affiliated Sixth People’s Hospital, Shanghai, 200233 China

**Keywords:** Ocular biometry, Cross-sectional study, Preschool children, Chinese

## Abstract

**Purpose:**

To investigate the distribution and changes in ocular biometry in 4-to to 9-year-old Chinese children and to compare the differences between age and genders in these parameters.

**Methods:**

This was a school-based cross-sectional study. A total of 1,528 Chinese children, aged 4–9 years, from one primary school and 12 kindergartens, were included in the study. Axial length, corneal curvature, anterior chamber depth, and corneal diameter were measured for each child.

**Results:**

AL and anterior chamber depth gradually increased with age in both genders. No significant changes in corneal curvature or corneal diameter were detected at different ages in either genders group. The mean ALs of males and females were 22.94 ± 0.80 mm and 22.38 ± 0.79 mm, respectively. The mean corneal curvatures of males and females were 43.05 ± 1.37 D and 43.75 ± 1.48 D, respectively. The mean anterior chamber depth of males and females were 3.47 ± 0.24 mm and 3.38 ± 0.25 mm, respectively. The mean corneal diameter of males and females were 12.08 ± 0.43 mm and 11.94 ± 0.44 mm, respectively. Females had consistently shorter ALs, shorter anterior chamber depth, smaller corneal diameter, and steeper corneal curvatures than males at any age.

**Conclusions:**

Boys had larger dimensions than girls for all ocular parameters except corneal curvature (flatter). Boys and girls showed similar trends for all parameters. Axial length and anterior chamber depth increased from 4 to 9 years of age, whereas corneal diameter and curvature did not change with age in either genders.

## Introduction

Myopia is a huge global health challenge [1, 2]. By 2050, the number of myopic patients is expected to reach nearly 5 billion, of whom 20% will suffer from high myopia [2, 3]. The global distribution of myopia is uneven, and the incidence of myopia in East Asia is significantly high [2]. At the same time, patients with high myopia have the potential risk of sight-threatening complications, such as myopic macular degeneration [3–5]. Myopic macular degeneration is one of the most common causes of irreversible loss of vision. In 2015, there were 10 million visually impairment and 3.3 million blind individuals worldwide [6–9]. Therefore, a key public health challenge is to identify the signs of myopia in children as soon as possible and conduct timely intervention or monitoring.

Normative values as a function of age are available for a variety of measurements such as height, weight and birthweight, and these values are a powerful tool used by clinicians for sensitively detecting aberrant growth at an early age. The shape of the eyeball may be a possible biomarker, descriptor, or risk factor for myopia, and measurements of the ocular components are essential in the study of myopia progression. Detailed documentation of ocular biometry parameters, such as axial length, corneal curvature, anterior chamber depth, and corneal diameter, will be helpful in identifying abnormalities during the emmetropization process in children.

Huang et al. [10] measured 651 Chinese children aged from 7 to 12 years, and found the mean spherical equivalent refractive error was + 0.26 ± 1.41 D, and axial length was 22.7 ± 0.90 mm. Huang et al. [11] measured 1,688 Chinese children aged from 36 to 48 months, and found statistically significant differences between the two eyes for the AL (21.88 mm in the right eyes; 21.86 mm in the left eyes, respectively) and corneal radius of curvature (CR) 1 (7.897 mm in the right eyes; 7.903 mm in the left eyes, respectively), whereas CR2 (7.657 mm in the right eyes; 7.657 mm in the left eyes, respectively) were similar. By comparison, Shimizu et al. [12] found that the children had steeper corneal curvature, longer inter–scleral spur distance, deeper anterior chamber than adults.

However, there are very little data on the eye biometrics of Chinese preschool children, especially those aged 4–6 years. Shanghai is located in the east of China, and is one of the biggest and the most important cities in China. Tianlin community belongs to Xuhui District, which is located in the center of Shanghai. The purpose of the present study was to investigate the distributions and changes in ocular biometry in 4-to to 9-year-old Chinese children and to compare the differences between age and genders in these parameters. We aim to provide basic data for the prevention and control of myopia in Shanghai and even the country.

## Methods

### Subjects

This school-based cross-sectional study was conducted in the Tianlin community of Shanghai, China from April 2019 to June 2019. There were eight kindergartens and three elementary schools in the Tianlin community. Children between four and nine years of age were recruited from one centrally located primary school and all eight kindergartens. The exclusion criteria were any serious eye disorders such as congenital cataract, glaucoma, uveitis, corneal pathologies, or premature retinopathy. Because 97.4% of the subjects were Han Chinese, and to eliminate any possible influence from different ethnic groups, only the results from Han Chinese children were reported. The study adhered to the Declaration of Helsinki and was approved by the Medical Research Ethics Committee of Shanghai 6th hospital. Informed consent was obtained from the parents or guardians of each child. All the parameters included in the manuscript were under Ethics approval and consent to participate section.

### Examination

All subjects underwent a full ophthalmic examination, including determination of best-corrected visual acuity (BCVA), slit-lamp examination, ophthalmoscopy, and intraocular pressure (IOP) testing using a noncontact tonometer (Nidek, Gamagori, Japan).

The axial length, corneal curvature, anterior chamber depth, and corneal diameter (white to white) of each eye were determined by an optometrist using an IOL Master 500 biometer (Carl Zeiss Meditec). The subject was positioned with the chin in a cup and the forehead against a headband. For each participant, five repeated sequential measurements were performed by one examiner in one eye. The corneal curvature was measured in the principal meridians to provide the greatest and least corneal curvatures. The mean corneal curvature was calculated using the average diopters of curvature.

It is well known that children’s compliance is poor, especially for children under 6 years of age. To increase compliance, the teacher accompanied the children during examinations. To decrease tension in children with poor compliance, the teacher would allow them to stand by and observe other children being measured. Five consecutive measurements were taken for each eye and the mean value was calculated to reduce the error.

### Calculations and statistics

All the data were entered into an Excel 2010 spreadsheet (Microsoft, Redmond, WA, USA) and SPSS software (SPSS Statistics 20.0; IBM, Armonk, NY, USA) was used for the statistical analyses. The distributions of ocular biometric parameters were tested for normality using the Kolmogorov-Smirnov test. The correlation between the right and left eyes was analyzed using Pearson’s correlation. Continuous variables are presented as mean ± standard deviation (SD). The Mann–Whitney U test or Kruskal–Wallis test was used to compare continuous data between two or three or more groups. All P-values were two-sided, and statistical significance was set at P < .05.

## Results

### Characteristics

A total of 1,558 children (854 males and 704 females), aged 4–9 years, were selected for this study. Sixteen subjects were not Han Chinese children; two had a history of penetrating corneal injury, and their results were excluded. Twelve participants showed poor compliance in the test. Several repeated measurements were performed; however, the data were incomplete. Therefore, their results were excluded. The coefficient of variation for all biometric measurements was less than 1%, and the average is used for subsequent analysis. Finally, 1528 children were included in the analysis. Table 1 presents the distribution of the 1528 subjects. As there were high Pearson correlations between the biometry data of the right and left eyes, ranging from 0.86 to 0.97, only the results for the right eye are presented.

**Table 1:** Distribution of the subjects

Age (years old)	Boys	Girls	Axial length (mm)	Corneal curvature (D)	Anterior chamber depth (mm)	Corneal diameter (mm)
4	71	79	22.19 ± 0.61	43.47 ± 1.41	2.82 ± 0.27	12.00 ± 0.43
5	275	248	22.43 ± 0.63	43.31 ± 1.27	2.87 ± 0.26	12.02 ± 0.43
6	279	205	22.65 ± 0.67	43.39 ± 1.31	2.91 ± 0.24	12.04 ± 0.44
7	83	47	22.76 ± 0.71	43.28 ± 1.57	2.93 ± 0.23	12.04 ± 0.41
8	49	55	23.24 ± 0.77	43.45 ± 1.50	3.00 ± 0.23	12.03 ± 0.47
9	67	70	23.64 ± 0.87	43.45 ± 1.48	3.11 ± 0.24	12.05 ± 0.42

### Axial length

The AL gradually increased with age in both genders. Significant changes were detected in both ganders (P < .01, and P < .01).Mean AL was 22.42 mm in boys and 21.92 mm in girls at 4 years old, 22.68 mm in boys and 22.14 mm in girls at 5 years old, 22.90 mm in boys and 22.36 mm in girls at 6 years old, 23.10 mm in boys and 22.35 mm in girls at 7 years old, 23.49 mm in boys and 22.92 mm in girls at 8 years old, and 23.83 mm in boys and 23.38 mm in girls at 9 years old. (Table 2)

**Table 2:** Summary of ocular biometry in children grouped by age and gender

Age (years old)	sex	Mean AL (mm)	P value	Mean CC (D)	P value	Mean AD (mm)	P value	Mean CD (mm)	P value
4	Male	22.42 ± 0.61	0.004	43.14 ± 1.42	0.002	3.34 ± 0.26	0.005	12.03 ± 0.44	0.007
	Female	21.92 ± 0.59		43.85 ± 1.40		3.27 ± 0.26		11.92 ± 0.43	
5	Male	22.68 ± 0.63	0.003	42.97 ± 1.31	0.003	3.40 ± 0.22	0.007	12.07 ± 0.45	0.003
	Female	22.14 ± 0.62		43.71 ± 1.16		3.31 ± 0.32		11.94 ± 0.41	
6	Male	22.90 ± 0.71	0.003	43.05 ± 1.17	0.003	3.44 ± 0.24	0.007	12.10 ± 0.41	0.003
	Female	22.36 ± 0.64		43.78 ± 1.42		3.35 ± 0.24		11.95 ± 0.48	
7	Male	23.10 ± 0.74	P < .001	42.94 ± 1.63	0.002	3.47 ± 0.24	P < .001	12.09 ± 0.42	0.004
	Female	22.35 ± 0.67		43.68 ± 1.50		3.34 ± 0.22		11.94 ± 0.41	
8	Male	23.49 ± 0.76	0.003	43.19 ± 1.35	0.007	3.53 ± 0.23	0.008	12.10 ± 0.46	0.004
	Female	22.92 ± 0.79		43.76 ± 1.74		3.44 ± 0.21		11.94 ± 0.46	
9	Male	23.83 ± 0.84	0.006	43.22 ± 1.16	0.007	3.64 ± 0.24	0.008	12.10 ± 0.43	0.006
	Female	23.38 ± 0.96		43.73 ± 1.79		3.55 ± 0.26		11.96 ± 0.40	

AL: aixal length; CC: corneal curvature; AD: anterior chamber depth; CD: corneal diameter

**Fig. 1:**
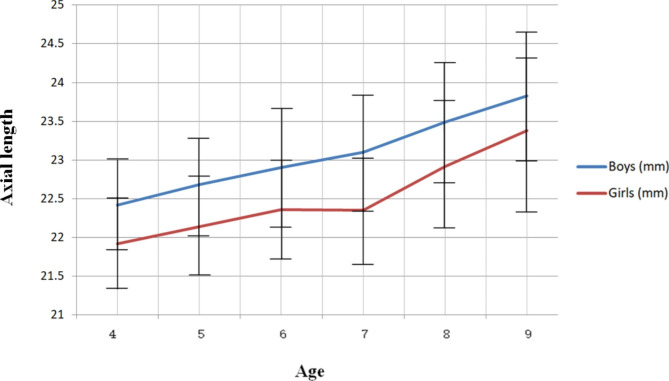
Axial length trend with age. The females had consistently shorter mean ALs than males at any age

The mean ALs of males and females were 22.94 ± 0.80 mm and 22.38 ± 0.79 mm, respectively. The females had consistently shorter mean ALs than males at any age. (Fig. 1) Between 4 and 7 years old, the average axial length growth was 0.22 mm/year in boys and 0.14 mm/year in girls, and between 7 and 9 years old, it was 0.36 mm/year in boys and 0.51 mm/year in girls. From 4 years old to 9 years old, the axial length totally elongated 1.41 mm in boys and 1.46 mm in girls.

The difference of the ALs between males and females (ALs in males minus ALs in females) was 0.50 mm at 4 years of age, 0.54 mm at 5 years of age, 0.54 mm at 6 years of age, 0.75 mm at 7 years of age, 0.57 mm at 8 years of age, and 0.45 mm at 9 years of age, respectively.

### Corneal curvature

Corneal curvatures are basically stable at different ages in either genders. No significant changes were detected in both genders (P > .05, and P > .05). Mean corneal curvature was 43.14 D in boys and 43.85 D in girls at 4 years old, 42.97 D in boys and 43.71 D in girls at 5 years old, 43.05 D in boys and 43.78 D in girls at 6 years old, 42.94 D in boys and 43.68 D in girls at 7 years old, 43.19 D in boys and 43.76 D in girls at 8 years old, and 43.22 D in boys and 43.73 D in girls at 9 years. (Table 2)

**Fig. 2:**
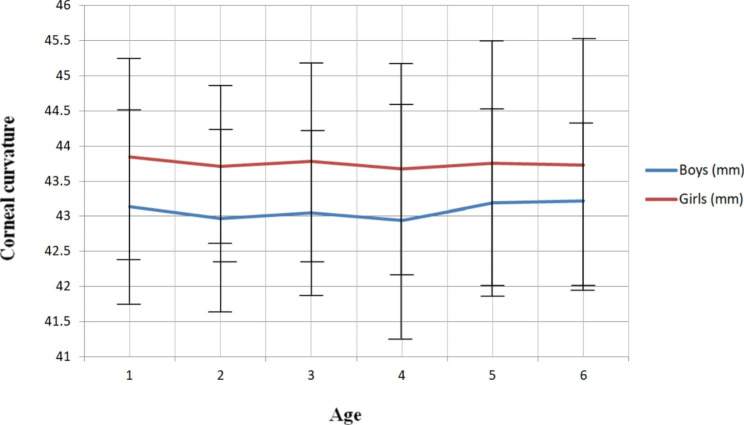
Corneal curvature trend with age. The females had consistently steeper mean corneal curvatures than males at any age

The mean corneal curvatures of males and females were 43.05 ± 1.37 D and 43.75 ± 1.48 D, respectively. Females consistently had steeper mean corneal curvatures than males at all ages. (Fig. 2) The difference of the corneal curvatures between males and females (corneal curvatures in females minus corneal curvatures in males) was 0.71 D at 4 years of age, 0.74 D at 5 years of age, 0.73 D at 6 years of age, 0.74 D at 7 years of age, 0.57 D at 8 years of age, and 0.51 D at 9 years of age, respectively.

### Anterior chamber depth

Anterior chamber depth gradually increased with age in both genders. Significant changes were detected in both genders (P < .01, and P < .01).Mean anterior chamber depth was3.34 mm in boys and 3.27 mm in girls at 4 years old, 3.40 mm in boys and 3.31 mm in girls at 5 years old, 3.44 mm in boys and 3.35 mm in girls at 6 years old, 3.47 mm in boys and 3.34 mm in girls at 7 years old, 3.53 mm in boys and 3.44 mm in girls at 8 years old, and 3.64 mm in boys and 3.55 mm in girls at 9 years. (Table 2)

**Fig. 3:**
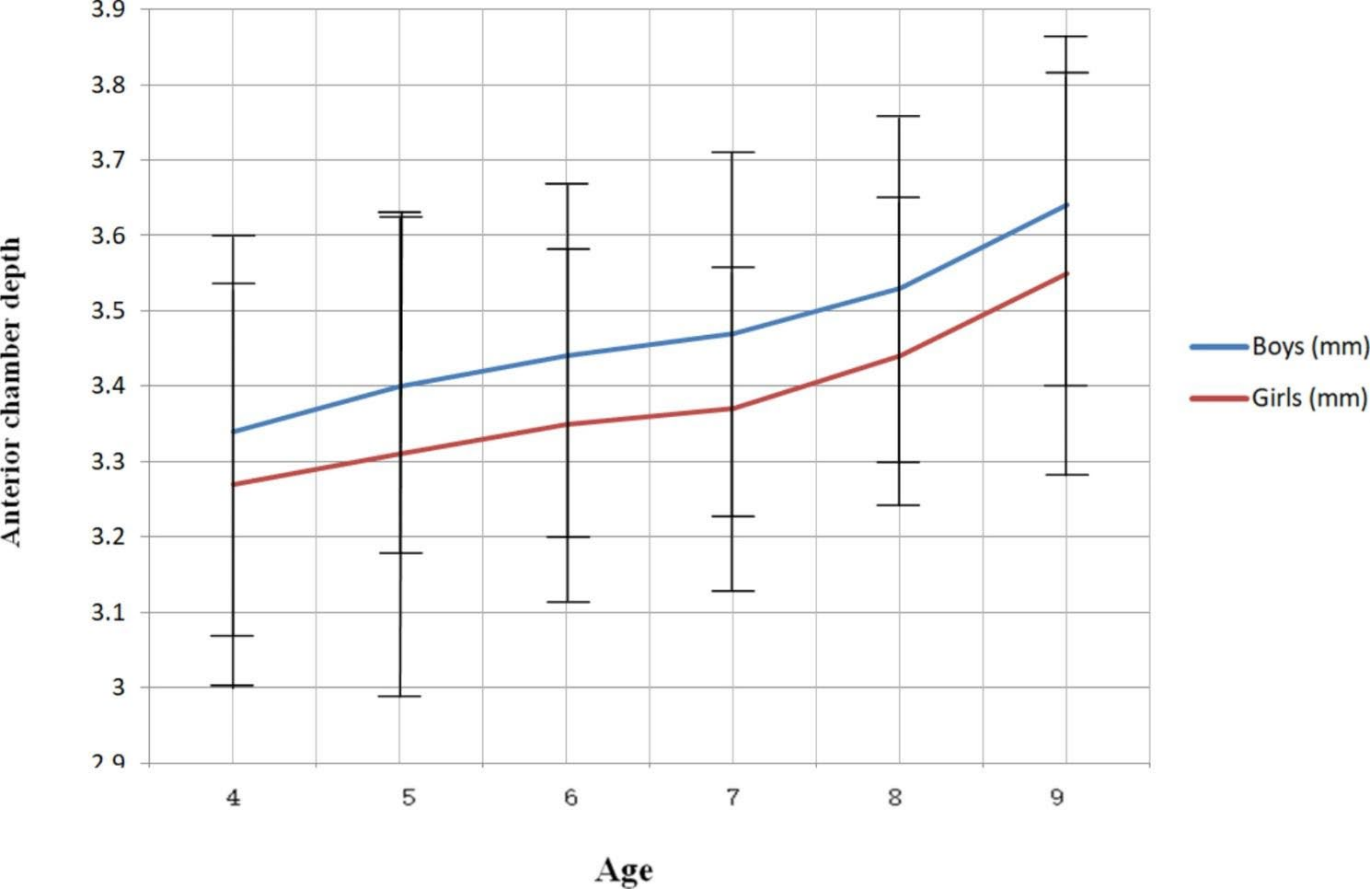
Anterior chamber depth trend with age. The females had consistently shorter mean anterior chamber depth than males at any age

The mean anterior chamber depth of males and females were 3.47 ± 0.24 mm and 3.38 ± 0.25 mm, respectively. The mean anterior chamber depth was consistently shorter in women than males at all ages. (Fig. 3) Between 4 and 7 years old, the average anterior chamber depth growth was 0.04 mm/year in boys and 0.02 mm/year in girls, and between 7 and 9 years old, it was 0.09 mm/year in boys and 0.11 mm/year in girls. From 4 years old to 9 years old, the anterior chamber depth totally elongated 0.30 mm in boys and 0.28 mm in girls.

The difference of the anterior chamber depth between males and females (anterior chamber depth in males minus anterior chamber depth in females) was 0.07 mm at 4 years of age, 0.09 mm at 5 years of age, 0.09 mm at 6 years of age, 0.13 mm at 7 years of age, 0.09 mm at 8 years of age, and 0.09 mm at 9 years of age, respectively.

### Corneal diameter

Corneal diameters are stable at different ages in either genders. No significant changes were detected in either genders (P > .05, and P > .05). Mean corneal curvature was 12.03 mm in boys and 11.92 mm in girls at 4 years old, 12.07 mm in boys and 11.94 mm in girls at 5 years old, 12.10 mm in boys and 11.95 mm in girls at 6 years old, 12.09 mm in boys and 11.94 mm in girls at 7 years old, 12.10 mm in boys and 11.94 mm in girls at 8 years old, and 12.10 mm in boys and 11.96 mm in girls at 9 years. (Table 2)

**Fig. 4:**
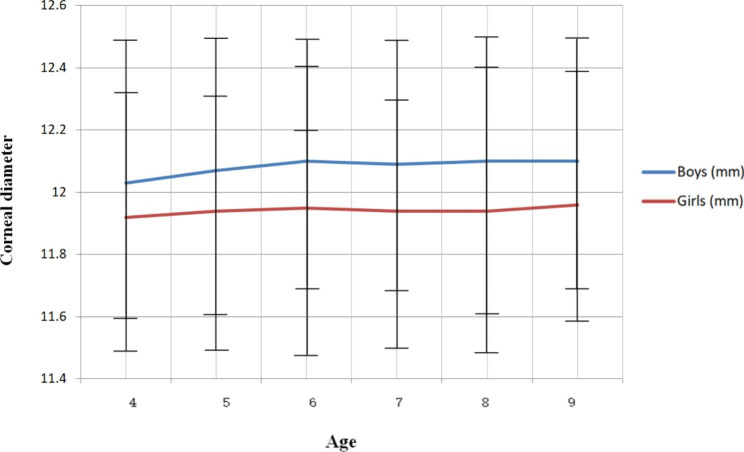
Corneal diameter trend with age. The females had consistently smaller mean corneal diameter than males at any age

The mean corneal diameter of males and females were 12.08 ± 0.43 mm and 11.94 ± 0.44 mm, respectively. The mean corneal diameter was consistently smaller in women than males at all ages. (Fig. 4) The difference of the corneal diameter between males and females (corneal diameter in males minus corneal diameter in females) was 0.11 mm at 4 years of age, 0.13 mm at 5 years of age, 0.15 mm at 6 years of age, 0.15 mm at 7 years of age, 0.16 mm at 8 years of age, and 0.14 mm at 9 years of age, respectively.

## Discussion

There were significant differences between the genders in all parameters. Measurements of axial length, anterior chamber depth, and corneal diameter were generally greater in boys than in girls. Corneal curvature measurements obtained from boys were flatter than those obtained from girls were. Boys and girls showed similar trends for all parameters. Axial length and anterior chamber depth increased from 4 to 9 years of age, whereas corneal diameter and curvature did not change with age in either genders.

Zhang et al [13]. reported that the mean ALs of children aged 3–6 years in Hebei Province, China was 22.31 mm (22.12 mm at age 4, 22.34 mm at age 5, and 22.49 mm at age 6). In this study, the mean ALs was 22.19 mm at age 4, 22.43 mm at age 5, 22.76 mm at age 6, which was longer than Hebei Province. A possible reason for the differences in the axial length could be the different environmental factors at different locations. However, differences in AL among different studies should also be treated with caution using different biometry instruments, such as IOLMaster versus ultrasound.

In our study, between 4 and 7 years of age, average AL increase was 0.22 mm/year in males and 0.14 mm/year in females, and between 7 and 9 years of age, average AL increase was 0.36 mm/year in males and 0.51 mm/year in females. The increase in ALs in subjects aged 7–9 years was greater than that in subjects aged 5–7 years for both genders. Meanwhile, at 7–9 years of age, the AL growth in females was greater than that in males. The exact reasons for these trends remain unclear, but differences in outdoor time may play a role. Children aged 7–9 years were enrolled in primary school. In kindergartens, the school schedule mainly includes playing, performing manual exercises, and reading chants, whereas in primary schools, the schedule mainly includes reading, writing, sports, and manual exercises. Li et al [14]. reported that males aged 10–15 years spent significantly more time outdoors (such as playing football, running, and riding a bicycle) and less time on homework than did females. Previous studies have reported that increasing outdoor activities can slow the rate of axial elongation in children [3, 15, 16].

We found that corneal curvature was relatively similar across age groups and that there were genders differences in corneal curvature. Females had a significantly steeper corneal curvature than males. Steeper corneal curvature in females has also been reported by previous authors in studies of children in the CLEERE study [17] and the Sydney myopia Study [18, 19]. These gender-related differences may be linked to the smaller size of the globe in young girls, which reach the value of axial length in boys by about 14 years of age [20].

The anterior chamber depth increased at the same rate in girls and boys aged 4–9 years. Rauscher et al [20]. suggested that an increase in AD is driven by two factors: a decrease in lens thickness and an increase in AL. Because of these two effects, AD increases up to 10 years of age, matching the decrease in LT. In our study, the girls presented with lower values of anterior chamber depth than boys (mean 0.09 mm shorter), which corresponds well with both Hashemi et al. ’s [21]. and Twelker et al. ’s [22] studies (0.1 mm shorter).

In Jiang et al’s [23] study on Chinese children aged 4 to 18 years, they found the mean horizontal corneal diameter was 12.0 ± 0.4 mm, which was correspondence with our study. In our study, no significant changes were detected in the corneal diameter at different ages in either genders. However, some previous studies have found that corneal diameter decreases with older age [24–26]. A possible reason for this inconsistency may be the small age range in our study, which may have caused insignificant changes with older age. Although our findings regarding corneal diameter distribution in the genders differed from those of Fu et al [27] our data are consistent with those of previous reports [25, 28, 29], in which male gender was associated with a larger corneal diameter.

Our study has some limitations. First, the analysis was based on cross-sectional rather than longitudinal data. It would be interesting to follow changes in these children’s ocular biometric parameters with age. Second, the children in our study were aged four or nine years; therefore, caution should be exercised when extrapolating these results to children of other ages. Third, we did not collect the refractive results of the children. This might cause skewing if a group of very astigmatic or myopic children have very different biometric characteristics.

In conclusion, our study presents the distribution of ocular biometry with age and genders variations in a large sample of four- and 9-year-old Chinese children. Boys had larger dimensions than girls for all ocular parameters except corneal curvature (flatter). Boys and girls showed similar trends for all parameters. Axial length and anterior chamber depth increased from 4 to 9 years of age, whereas corneal diameter and curvature did not change with age in either genders.

## Data Availability

The datasets used and analysed during the current study available from Ming-hui Zhao (E-mail address: zhao_m_h@163.com) on reasonable request.
